# Factors associated with SARS-CoV2 infection and care pathways among the most vulnerable populations living in Marseille: a case control study

**DOI:** 10.1186/s12889-021-11716-6

**Published:** 2021-09-19

**Authors:** Ismaïl Alsaïdi, Frédéric De Sousa Santos, Bérengère Plard, Elise Janvier, Aurélie Tinland, Abdelmajid Hafni, Emilie Mosnier

**Affiliations:** 1 Aix-Marseille University, School of Medicine – La Timone Medical Campus, Marseille, France; 2 Association d’Aide aux Jeunes Travailleurs, Marseille, France; 3 CEReSS – Health Service Research and Quality of Life Center, Marseille, France; 4 Alphabio, Marseille, France; 5 Centre Hospitalier d’Alès-Cévenne, Alès, France; 6 INSERM, IRD, SESSTIM, ISSPAM, Marseille, France

**Keywords:** COVID-19, Risk factors, Vulnerable populations, Social determinants of health, Healthcare disparities, Healthcare delivery

## Abstract

**Background:**

The Covid-19 pandemic has led to substantial and unexpected increases in morbidity and mortality in France. Vulnerable populations housed in accommodation centres have a greater risk of infection because collective housing and their dependence on social support services mean it is more difficult to apply preventive measures. They are also at greater risk of developing severe forms of Covid-19 and waiting longer before seeking healthcare (for Covid-19 or other) treatment. We aimed to identify the factors associated with SARS-CoV2 infection in the most vulnerable populations in the city of Marseille.

**Methods:**

The study sample comprised users of various services provided by the association AAJT in Marseille, France, some presenting symptoms suggestive of Covid-19 and others not. All had routine health surveillance provided by AAJT’s dedicated healthcare team between March 2020 and May 2020. Using univariate and multivariate analyses, we studied the influence of several variables on morbidity associated with Covid-19.

**Results:**

The study included 64 participants, 29 of whom tested positive for Covid-19 and 35 control subjects. Median age was 21.16 years old. Individuals in the ‘Covid-19 case’ group (*p* < 0.005) – which included persons testing positive and those suspected of being infected – were younger. The study sample’s male/female ratio was seven. In our multivariate analyses, living in a shared apartment and poor adherence to social distancing measures were factors associated with Covid-19 infection. Furthermore, mental health problems - such as anxiety disorder - were very frequent in the study sample.

**Conclusions:**

Allocating more and specific housing units to structures providing accommodation services to the most vulnerable people would seem to be a decisive factor in controlling the spread of SARS-CoV2, and deserves more attention from public authorities.

**Supplementary Information:**

The online version contains supplementary material available at 10.1186/s12889-021-11716-6.

## Background

During the first half of 2020, the whole world was plunged into an unprecedented health crisis due to the emergence of a novel Coronavirus - SARS-CoV2 - responsible for Covid-19 (*Corona Virus Disease 2019*) [1]. The initial epidemic started in China at the end of December 2019, in the city of Wuhan (Hubei province). At the time of writing (April 2021), the infection is continuing its exponential spread in some regions, and millions of cases have already been detected worldwide [2, 3]. The pandemic has resulted in substantial and unexpected increases in morbidity and mortality in Europe and mainland France [4, 5]. Elderly people, and those with co-morbidities appear to be at greater risk of morbidity and mortality [6].

People living in social precarity hosted in accommodation centres are at particular risk of infection [7]. Their dependence on the support system, together with collective housing where they stay, make it hard for them to adequately follow government safety recommendations and apply Covid-19 preventive measures [8]. Furthermore, because of their difficult lifestyles, they are possibly at greater risk of more severe forms of Covid-19 and wait longer before seeking medical treatment, whether for Covid-19 or other reasons [9, 10]. Moreover, the resulting international economic crisis which the current pandemic has created together with associated indirect consequences (e.g., risk of malnutrition, sleeping disorders), have greatly worsened these populations’ health [11]. These two issues highlight the need for suitable measures to ensure these vulnerable people are provided safe accommodation [12]. Our study aimed to identify the possible factors associated with SARS-CoV2 infection in people living in social deprivation and who are provided accommodation in various structures in Marseille. Our study’s secondary objective was to describe their care pathways in the current pandemic context. To test our hypotheses, our investigation field was the non-governmental organization (NGO) ‘Aid to Young Workers Association’ (AAJT).

Since 1954, the AAJT has helped provide support services to approximately 800 people (minors and adults) every year from vulnerable populations in the city of Marseille in southern France and the surrounding area. Inter alia, the AAJT provides collective accommodation which, in the current context, is a factor for increased SARS-CoV2 transmission [13]. The AAJT is aware of the crucial role that a patient’s healthcare pathway plays in quality of life and of the negative consequences of refusing healthcare opportunities. Since January 2018, it has implemented a regional programme for access to general disease prevention and healthcare services for these populations, entitled PRAPS. Overseen by a dedicated healthcare team (comprising caregivers) within the association’s structure, the programme provides assistance to users of its services including support for patient healthcare pathways, training, prevention activities, and skills in health self-management. Mapping the region’s existing healthcare network is also a crucial element in the programme. In the current health crisis context, AAJT’s healthcare team are paying particular attention to patients presenting symptoms suggestive of Covid-19 and are playing a crucial role in managing psychological issues in the youngest and most vulnerable users of the organization’s services [14].

## Methods

This is a retro-prospective case-control observational study conducted between March 2020 and May 2020.

### Study design and participants

Our study focused on vulnerable populations, mainly migrants and homeless people, living in Marseille and provided accommodation by various organisations, including the AAJT. The AAJT provides accommodation to hundreds of users every year [15]. The study sample for our present study comprised users of AAJT’s services, some with symptoms suggestive of Covid-19 and some without, all of whom were routinely provided healthcare surveillance by the association’s dedicated health team between March and May 2020. This time period overlaps with the timing of the first national Covid-19-related lockdown in France. Our study participants were users of eight different support services provided by AAJT as follows: support service for young adults; reception centre for asylum seekers; social accommodation for young workers; social integration facilities; emergency accommodations; collective social housing for children; geographically diffuse social housing for children; professional & social inclusion facilities. As can be seen from this list, some of the study participants were staying in accommodation provided by AAJT, while others were staying in accommodation provided by other structures in Marseille.

Study eligibility criteria were as follows: being monitored by AAJT’s dedicated healthcare team between March 2020 and May 2020, staying in social accommodation within the perimeter of the city of Marseille, and providing informed consent to data being used in the context of the study.

The choice to implement this study exclusively with AAJT’s service users was justified by the large number of people who benefit annually from its services, and the fact that the organisation provides healthcare services and monitoring to all its users.

### Data collection

Data were collected from AAJT’s available databases and from a standardized questionnaire based on the French National Authority for Health recommendations which was specifically modified to match with the study objectives [16]. This pseudonymised questionnaire collected the following data: socio-demographic characteristics, recent life history, medical history and comorbidities, treatments, clinical-biological and medical follow-up data. [Additional file 1] The questionnaires were completed by the AAJT healthcare team to ensure a familiar environment for study participants and to minimize social desirability bias [17].

Following the Covid-19 mitigation policy adopted by France in 2020, each AAJT service user presenting symptoms suggestive of the disease had to quarantine for 14 days and received both remote (e.g., telephone call with general practitioner) and home-based (e.g., visits from a nurse) medical follow-up on Day1 (D1), D3, D5, D9 and D14 after the onset of symptoms [18].

### Biological analysis

An RT-PCR (nasopharyngeal swab) for SARS-CoV2 screening campaign ran in all the eight AAJT services listed above between 4 May and 15 May 2020, and was open to all the organisation’s users. RT-PCR samples were taken by a medical team comprising nurses and doctors. During three of the campaign’s numerous sessions, the healthcare team submitted the questionnaire to several randomly selected users. Rapid diagnostic orientation serological tests (BIOSYNEX®) for SARS-CoV2 were also performed in all AAJT structures in two screening sessions organized on 24 June and 1 July 2020. Data on study participants who had a rapid test were also collected.

### Covid-19 case definitions

To assess each of our study participants’ vulnerability, we used the High Council for Public Health’s (HCSP) definition of social deprivations follows: ‘a state of social instability characterized by a loss of security’. [19].

From the clinical and biological data gathered, four distinct categories were formed [20] as follows:

**Confirmed Covid-19 cases:** *Any patient with a positive SARS-CoV2 PCR test result and/or positive rapid diagnostic serological orientation test result.*

**Suspected Covid-19 cases:** *Any patient presenting a clinical situation and/or chest scan suggestive of COVID-19 symptoms, but with no SARS-CoV2 PCR test or no rapid diagnostic serological orientation test taken.*

**Users not suspected of having Covid-19:** *Patients not symptomatic with no screening test (PCR or rapid serological) taken.*

**Users identified as not having Covid-19**: *Patients not symptomatic and with a negative SARS-CoV2 PCR result and/or a negative rapid diagnostic serological orientation test result.*

As per French guidelines, a patient was considered to have symptoms suggestive of Covid-19 when they presented with a fever (> 37 °C) and either a cough or two other Covid-19-like symptoms (headache, asthenia, dyspnoea, chest pain, anosmia, myalgia) [21]. All persons suspected of being Covid-19 positive were medically examined for confirmation.

In order to have two groups to compare for this case-control study, we combined the ‘confirmed’ and ‘suspected’ categories to create a ‘Covid-19 cases’ group, and the ‘not suspected’ and ‘identified as not having Covid-19’ categories to create a control group.

Duration was considered when assessing symptom severity [22]. Four forms of Covid-19 (reflecting different levels of disease severity) were described as follows:

**Asymptomatic:** *No symptom suggestive of Covid-19 infection.*

**Benign:** *Mild symptoms and intensity greatly reduced before D5.*

**Moderate:** *Moderate symptoms and intensity greatly reduced before D9.*

**Severe:** S*evere symptoms and/or several symptoms and/or requiring hospitalization.*

Finally, we considered that an individual was at risk of contracting a severe form of Covid-19 when they had one or more known risk factors *or* were a carrier of tuberculosis *or* had multimorbidity [23–27].

### Statistical analyses

The primary outcome was morbidity associated with SARS-CoV-2 infection.

We performed univariate and multivariate analyses (Covid-19 case group vs. control group). Depending on the explanatory variable considered, several tests were used to perform the univariate analyses as follows: the Wilcoxon’s Rank sum test for continuous variables with small samples, the χ^2^ test for categorical variables with large samples, and Fisher’s exact test for categorical variables with small samples. The Kaplan-Meir estimator with the log-rank test was used to measure the differences between our participants regarding the amount of time before they were provided medical assistance after symptom onset (Temporal variable).

Logistic regression was chosen to conduct the multivariate analyses because of the heterogeneity in the variables evaluated. We used a step-down procedure to select the variables to include in our multivariate models. We chose to keep the model characterized by the lowest Akaike information criterion. A *p*-value < 0.05 was considered significant.

All the analyses and graphics were performed using R software (v. 3.6.3).

## Results

**Fig. 1:**
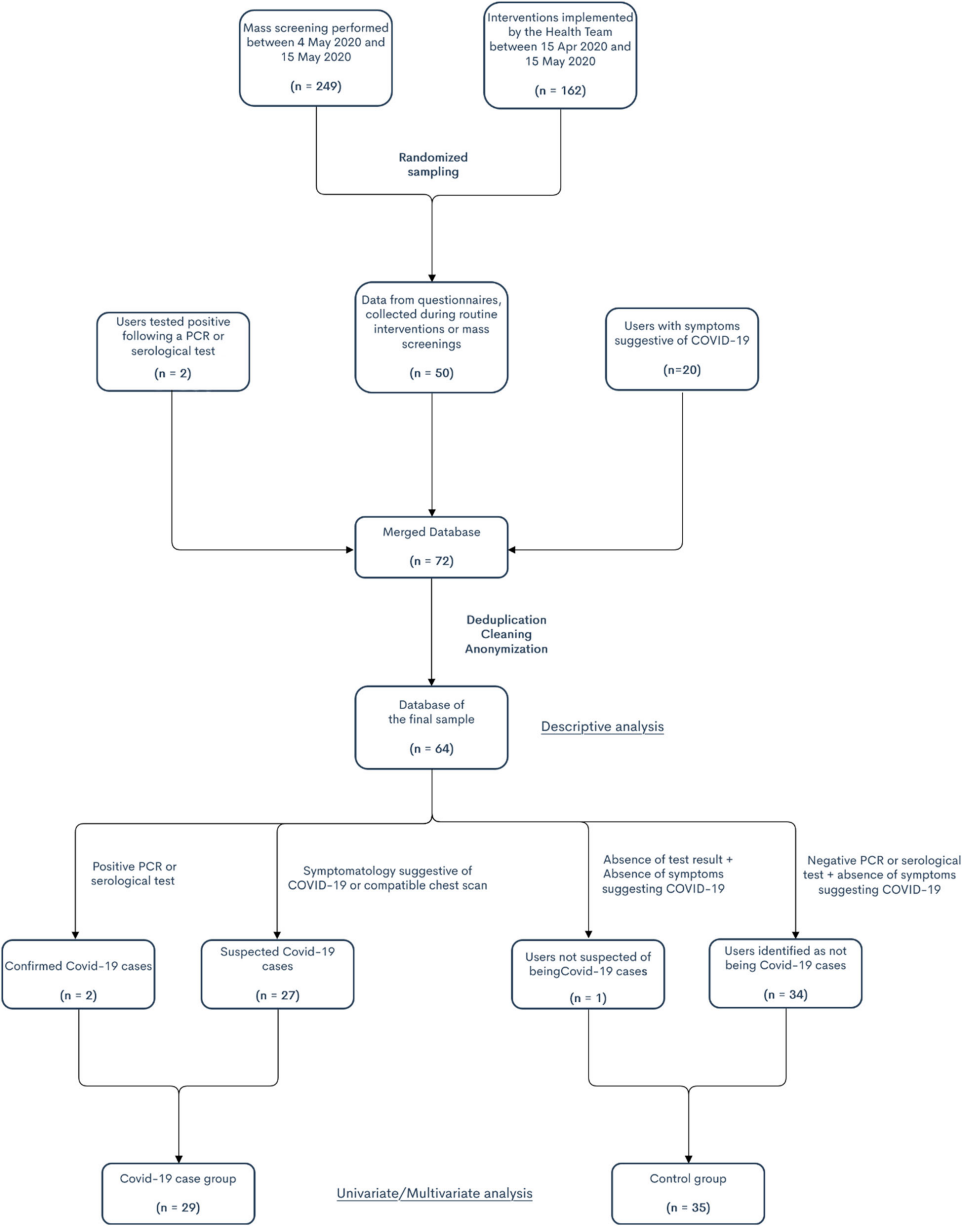
Flowchart of study recruitment and main sampling results

Sixty-four AAJT accommodation service users were included in our study. Of these, 29 were in the ‘Covid-19 cases’ group and 35 in the control group shows the number of users within the eight different services included (Fig. 1,Table 1). Table 2 summarizes participants’ socio-demographic characteristics, Table 3 their psycho-social situation during France’s first lockdown, Table 4 their adherence to Covid-19 preventive measures, and Table 5 the measures implemented to stop the spread of Covid-19.

**Table 1:** Number of users in each of the eight AAJT care services studied and according to study group

AAJT services	***Users (n = 64)***	Covid-19 case group (***n*** = 29)	Control group (***n*** = 35)
***Support for young adults***	***3.45% (1)***	8.57% (3)	6.25% (4)
***Reception Centre for Asylum Seekers***	***13.79% (4)***	2.86% (1)	7.81% (5)
***Social Residence for Young Workers***	***13.79% (4)***	20% (7)	17.19% (11)
***Social integration facilities***	***10.35% (3)***	31.43% (11)	21.88% (14)
***Emergency accommodations***	***6.90% (2)***	11.43% (4)	9.38% (6)
***Social Housing for Children (Collective)***	***10.35% (3)***	5.71% (2)	7.81% (5)
***Social Housing for Children (Diffuse)***	***37.93% (11)***	14.29% (5)	25% (16)
***Professional & social inclusion facilities***	***3.45% (1)***	5.71% (2)	4.69% (3)

*AAJT: Association for Helping Young Workers

Median age of the study population was 21.16 years (CI_95%_ = [20.10; 22.22]) and was lower in the ‘Covid-19 case’ group (*p* < 0.005).(Table 2) The majority (87.50%, *n* = 56/64) were men (male/female ratio = 7). Most participants were single (87.50%, n = 56/64) and had no dependent children (90.63%, *n* = 58/64). A majority had either a junior-high school education level (54.9%, *n* = 35/64) or no educational diploma (32.81%, *n* = 21/64). A substantial portion were either students (54.69%, n = 35/64) or did not practice a professional activity (20.31%, *n* = 13/64). More than half came from West Africa (53.13%, *n* = 34/64). One fifth were from the Middle East (18.75%, *n* = 12/64). The remaining participants (28.12%, *n* = 28/64) came from other parts of Africa, Europe and overseas French territories. The average time the participants had lived in France was 5.37 years (CI_95%_ = [3.43; 7.31]). They were provided different types of accommodation in the various structures, slightly more than half having single rooms (54.69%, *n* = 35/64). Only 2 of the 51 who had a PCR test during the screening campaign, tested positive. All 10 participants who had rapid serological tests tested negative. In terms of substance addiction, some users reported regular consumption of tobacco, alcohol or cannabis, but none reported using hard drugs use. Taking into account psychoactive substance consumption, the presence of chronic diseases and multimorbidity, the overall percentage of users at risk of developing a severe form of COVID-19 was 51.56% (*n* = 33/64).

### Care pathways

Almost all participants received social care benefits (96.88%, *n* = 62/64), with a large majority receiving routine benefits (i.e., national health insurance cover, complementary insurance cover, etc.) (89.06%, *n* = 57/64) (Table 2). State Medical Assistance refers to a special type of social support in France reserved for people in an irregular administrative situation (e.g., no work permit) and asylum seekers.

**Table 2:** Description of study participants’ socio-demographic characteristics according to study group

Variable	***Modality***	***Users (n = 64)***	Covid-19 case group (***n*** = 29)	Control group (***n*** = 35)	***Univariate analysis*** ***(***α = 0.05)
**Sex**	***Male***	***87.5% (56)***	82.76% (24)	91.43% (32)	*p* = 0.451
	***Female***	***12.5% (8)***	17.24% (5)	8.57% (3)	
**Age**		***m = 21.16 years***	m = 19.69 years	m = 22.55 years	***p*** **= 0.005935**
		***s = 4.34 years***	s = 4.96 years	s = 3.47 years	
**Geographical area of origin**	***Central Africa***	***1.57% (1)***	0% (0)	2.86% (1)	*p* = 0.8263
	***East Africa***	***3.13% (2)***	0% (0)	5.71% (2)	
	***North Africa***	***7.81% (5)***	10.35% (3)	5.71% (2)	
	***West Africa***	***53.13% (34)***	51.72% (15)	54.29% (19)	
	***Middle-East***	***18.75% (12)***	20.69% (6)	17.14% (6)	
	***East Europe***	***1.57% (1)***	3.45% (1)	0% (0)	
	***West Europe***	***1.57% (1)***	3.45% (1)	0% (0)	
	***France***	***7.81% (5)***	6.90% (2)	8.57% (3)	
	***Overseas French territories***	***4.69% (3)***	3.45% (1)	5.71% (2)	
**Time living in France**		***m = 5.37 years***	m = 4.38 years	m = 6.06 years	*p* = 0.06344
		***s = 7.52 years***	s = 7.23 years	s = 7.75 years	
**Education level**	***No diploma***	***32.81% (21)***	41.38% (12)	25.71% (9)	***p*** **= 0.04083**
	***Junior high school***	***54.69% (35)***	58.62% (17)	51.43% (18)	
	***High school***	***7.81% (5)***	0% (0)	14.29% (5)	
	***University***	***4.69% (3)***	0% (0)	8.57% (3)	
**Type of occupation**	***No professional activity***	***20.31% (13)***	31.04% (9)	11.43% (4)	***p*** **= 0.01304**
	***Student***	***54.69% (35)***	62.07% (18)	48.57% (17)	
	***Factory worker***	***1.56% (1)***	0% (0)	2.86% (1)	
	***Artisan***	***6.25% (4)***	3.45% (1)	8.57% (3)	
	***Employee***	***17.19% (11)***	3.45% (1)	28.57% (10)	
**Type of accommodation**	***Single room***	***54.69% (35)***	44.83% (13)	62.86% (22)	***p*** **= 0.0002112**
	***Single apartment***	***10.94% (7)***	17.24% (5)	5.71% (2)	
	***Shared room***	***17.19% (11)***	3.45% (1)	28.57% (10)	
	***Shared apartment***	***17.19% (11)***	34.48% (10)	2.86% (1)	
**Conjugal status**	***Single***	***87.50% (56)***	82.76% (24)	91.43% (32)	*p* = 0.451
	***In a relationship***	***12.50% (8)***	17.24% (5)	8.57% (3)	
**Number of dependent children**	***None***	***90.63% (58)***	89.66% (26)	91.43% (32)	*p* ≈ 1
	***1***	***6.25% (4)***	6.90% (2)	5.71% (2)	
	***2***	***3.12% (2)***	3.45% (1)	2.86% (1)	
**Social support**	***No social support***	***3.12% (2)***	3.45% (1)	2.86% (1)	*p* ≈ 1
	***State Medical Assistance***	***7.81% (5)***	6.90% (2)	8.57% (3)	
	***Routine social support (*i.e.*, national health insurance cover, complementary insurance cover,*etc.*)***	***89.06% (57)***	89.65% (26)	88.57% (31)	

*AAJT: Association for Helping Young Workers

Almost a quarter of the participants reported being hospitalized sometime between January 2020 and May 2020 (23.44%, *n* = 15/64). For those in the ‘Covid-19 case’ group, the average delay between their most recent hospitalisation and the onset of symptoms was 59.64 days (CI_95%_ = [51.68; 67.60]). A large proportion of participants had been screened for tuberculosis in 2019 or 2020 (61.02%, *n* = 36/59), with only 5.56% (2/36) testing positive. The percentage difference in terms of positive tuberculosis screening was not significant between the ‘Covid-19 case’ group and the control group.

**Fig. 2:**
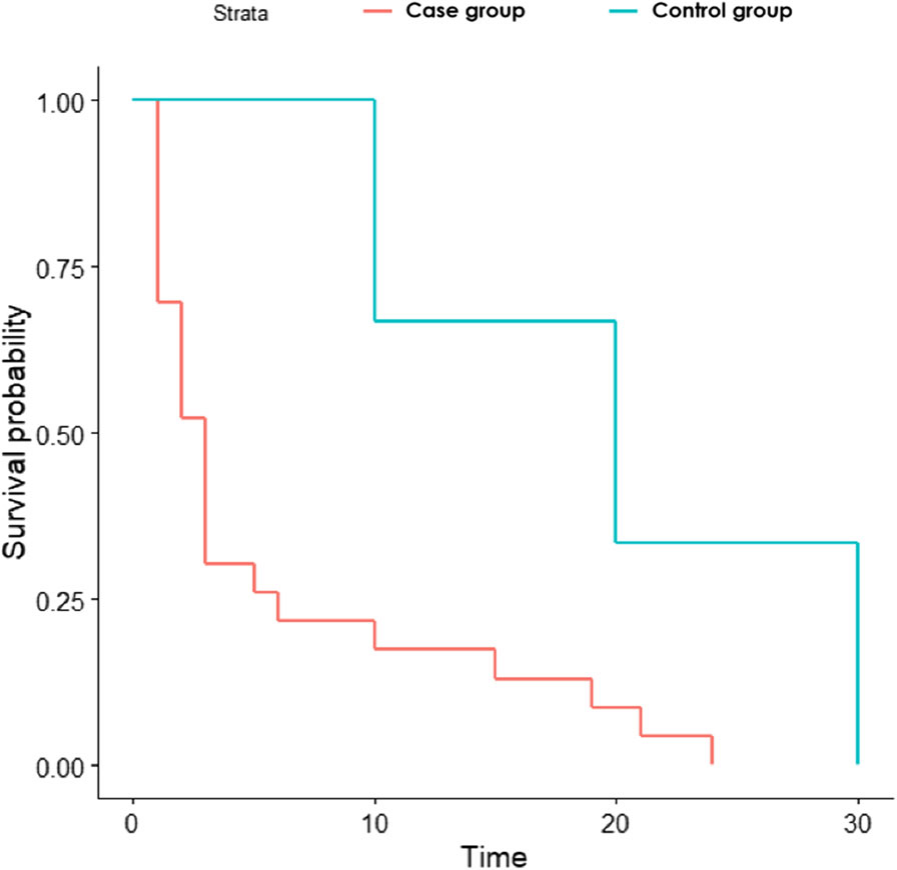
Delay between onset of symptoms (suggestive of Covid-19 or not) and access to medical treatment: Covid-19 case group vs. control group (Kaplan-Meier curves)

A significant portion of participants had experienced sadness (32.81%, *n* = 21/64), anxiety (42.19%, *n* = 27/4) or insomnia (32.81%, n = 21/64). Univariate analyses highlighted that the ‘Covid-19 case’ group tended to experience anxiety more than those in the control group before the onset of the first Covid-19 symptoms. However, a large proportion of the AAJT’s scheduled psychological counselling sessions were maintained for the participants during France’s first lockdown (32.81%, *n* = 21/64) (Table 3). Furthermore, hetero-aggressive behaviour and adherence to social distancing measures were correlated with an odds ratio significantly lower than 1. There were fewer reported reasons for consulting the AAJT healthcare team with respect to before the pandemic, but the reasons were still varied despite the lockdown. We observed a significant difference in the delay between symptom onset and healthcare between users presenting symptoms suggestive of Covid-19 (5.65 days) and others (20 days) (Fig. 2). Just over half of all the participants (51.56%, *n* = 33/64) were at risk of contracting a severe form of Covid-19 because they had a chronic pathology or multimorbidity. Nevertheless, no severe form was observed in any of the 64 participants included in the present study. With regard to adherence to preventive measures, 60.9% (*n* = 39/64) of participants wore a mask, 79.69% (51/64) socially distanced themselves, 85.94% (*n* = 55/64) regularly washed their hands, and 75% (*n* = 48/64) complied with lockdown measures (Table 4).

**Table 3:** Psycho-social situation of participants during France’s first lockdown (March 2020–May 2020) according to study group

Variable	***Modality***	***Users (n = 64)***	Covid-19 case group (***n*** = 29)	Control group (***n*** = 35)	***Univariate analysis*** ***(***α = 0.05)
**Psychological support**	***Yes***	***32.81% (21)***	34.48% (10)	31.43% (11)	*p* ≈ 1
	***No***	***67.19% (43)***	65.52% (19)	68.57% (24)	
**Decompensation observed in the participant**	***Yes***	***4.69% (3)***	6.90% (2)	2.86% (1)	
	***No***	***95.31% (61)***	93.10% (27)	97.14% (34)	
**Anxiety**	***Yes***	***42.19% (27)***	62.07% (18)	25.71% (9)	***p*** **= 0.007421**
	***No***	***57.81% (37)***	37.93% (11)	74.29% (26)	
**Sadness**	***Yes***	***32.81% (21)***	41.38% (12)	31.43% (9)	*p* = 0.2886
	***No***	***67.19% (43)***	58.62% (17)	68.57% (26)	
**Insomnia**	***Yes***	***32.81% (21)***	24.14% (7)	40% (14)	*p* = 0.2811
	***No***	***67.19% (43)***	75.86% (22)	60% (21)	
**Delirium observed in the participant**	***Yes***	***1.56% (1)***	3.45% (1)	0% (0)	*p* = 0.4531
	***No***	***98.44% (63)***	96.55% (28)	100% (35)	
**Hetero-aggressive behaviour observed in the participant**	***Yes***	***10.94% (7)***	20.69% (6)	2.86% (1)	***p*** **= 0.0401**
	***No***	***89.06% (57)***	79.31% (23)	97.14% (34)	
**Self-aggressive behaviour observed in the participant**	***Yes***	***1.56% (1)***	3.45% (1)	0% (0)	*p* = 0.4531
	***No***	***98.44% (63)***	96.55% (28)	100% (35)	
**Overdose observed in the participant**	***Yes***	***4.69% (3)***	6.90% (2)	2.86% (1)	*p* = 0.5859
	***No***	***95.31% (61)***	93.10% (27)	97.14% (34)	
**Nutrition**	***Sufficient/Healthy***	***66.56% (49)***	69.97% (20)	82.86% (29)	*p* = 0.3127
	***Insufficient/Unhealthy***	***23.44% (15)***	31.03% (9)	17.14% (6)	
**Sleep**	***Sufficient/Healthy***	***53.97% (34)***	57.14% (16)	51.43% (18)	*p* = 0.8432
	***Insufficient/Unhealthy***	***46.03% (29)***	42.86% (12)	48.57% (17)	
**Body hygiene**	***Sufficient/Healthy***	***89.06% (57)***	75.86% (22)	100% (35)	***p*** **= 0.002512**
	***Insufficient/Unhealthy***	***10.94% (7)***	24.14% (7)	0% (0)	
**Physical activity**	***Sufficient/Healthy***	***64.06% (41)***	58.62% (17)	68.57% (24)	*p* = 0.5726
	***Insufficient/Unhealthy***	***35.94% (23)***	41.38% (12)	31.43% (11)	
**Social life**	***Sufficient/Healthy***	***59.38% (38)***	51.72% (15)	65.71% (23)	*p* = 0.3795
	***Insufficient/Unhealthy***	***40.62% (26)***	48.28% (14)	34.29% (12)	

*AAJT: Association for Helping Young Workers

**Table 4:** Variables of infection risk and adherence to Covid-19 preventive measures (between January 2020 and May 2020) both overall and according to study group

Variable	***Modality***	***Users (n = 64)***	Covid-19 cases group (n = 29)	Control group (n = 35)	***Univariate analysis*** ***(***α = 0.05)
**Wearing a mask**	***Yes***	***60.94% (39)***	48.28% (14)	71.43% (25)	*p* = 0.1026
	***No***	***39.06% (25)***	51.72% (15)	28.57% (10)	
**Compliance with lockdown measures**	***Yes***	***75% (48)***	62.07% (18)	85.71% (30)	*p* = 0.05947
	***No***	***25% (16)***	37.93% (11)	14.29% (5)	
**Compliance with social distancing measures**	***Yes***	***79.69% (51)***	62.07% (18)	94.29% (33)	***p*** **= 0.004016**
	***No***	***20.31% (13)***	37.93% (11)	5.71% (2)	
**Regular hand washing**	***Yes***	***85.94% (55)***	75.86% (22)	94.29% (33)	*p* = 0.06688
	***No***	***14.06% (9)***	24.14% (7)	5.71% (2)	
**Access to hydroalcoholic solution to wash hands**	***Yes***	***50% (32)***	48.28% (14)	51.43% (18)	p ≈ 1
	***No***	***50% (32)***	51.72% (15)	48.57% (17)	
**Presence of persons suspected of having Covid-19: family/close friends**	***Yes***	***17.19% (11)***	37.93% (11)	0% (0)	
	***No***	***82.81% (53)***	62.07% (18)	100% (35)	
**Hospitalisation**	***Yes***	***23.44% (15)***	20.69% (6)	25.71% (9)	*p* = 0.8603
	***No***	***76.56% (49)***	79.31% (23)	74.29% (26)	
**Time between last hospital visit and the onset of symptoms suggestive of Covid-19**		***m = 59.64 days***	m = 36.67 days	m = 76.88 days	***p = 0.02***
		***s = 32.50 days***	s = 30.94 days	s = 22.05 days	
**Participant compliance with self-isolation requirements**	***Yes***		***65% (13)***		
	***No***		***35% (7)***		

*AAJT: Association for Helping Young Workers

Among participants in the ‘Covid-19 case’ group, 20.69% (*n* = 6/29) were asymptomatic cases, 51.72% (*n* = 15/29) had a mild form of the disease, and 27.59% (8/29) a moderate form. A majority of symptomatic users had received Covid-19-related medical advice (78.26%, *n* = 18/23), treatment (86.96%, *n* = 20/23) and single accommodation guaranteeing strict isolation (82.61%, *n* = 19/23). A relatively large share of the 64 study participants had a PCR test during the scheduled campaigns (79.69%, *n* = 51/64), a smaller share having the rapid serological test (15.63%, *n* = 10/64) screening sessions. PCR tests were performed on average 28.29 days after the onset of symptoms. Only 6.9% (*n* = 2/29) of participants in the ‘Covid-19 case’ group had a chest scan. Furthermore, only 65% ​​(*n* = 13/20) of potential cases complied with lockdown measures (Tables 4 and 5).

**Table 5:** Measures implemented to stop the spread of Covid-19

Variable	***Modality***	***Users (n = 64)***	Covid-19 case group (n = 29)	Control group (n = 35)	***Univariate analysis*** ***(***α = 0.05)
**Time between symptom onset and medical consultation**		***m = 7.31 days***	m = 5.65 days	m = 20 days	**p = 0.04**
		***s = 8.59 days***	s = 7.06 days	s = 10 days	
**Denial about the possibility of being Covid-19 positive**	***Yes***		***39.13% (9)***		
	***No***		***60.87% (14)***		
**Strict self-isolation measures for confirmed and suspected participants**	***Yes***		***82.61% (19)***		
	***No***		***17.39% (4)***		
**Modification of accommodation type for the participant following confirmation of Covid-19 positivity**	***Yes***		***8.70% (2)***		
	***No***		***91.30% (21)***		
**Hospitalization following the onset of symptoms suggestive of Covid-19**	***Yes***		***4.35% (1)***		
	***No***		***95.65% (22)***		
**Prescription of medicines to treat infection**	***Yes***		***86.96% (20)***		
	***No***		***13.04% (3)***		
**Duration of symptoms suggestive of Covid-19**	***1–2 days***		***17.39% (4)***		
	***2–3 days***		***21.74% (5)***		
	***3–5 days***		***13.04% (3)***		
	***5–9 days***		***30.44% (7)***		
	***9–14 days***		***17.39% (4)***		
**Time between the onset of symptoms and nasopharyngeal PCR test**			***m = 28.29 days***		
			***s = 23.82 days***		
**PCR test results**	***Positive***	***3.92% (2)***			
	***Negative***	***96.08% (49)***			
**Serological test results**	***Positive***	***0% (0)***			
	***Negative***	***100% (10)***			
**Conclusions regarding COVID status**	***Identified as not having Covid-19***	***53.13% (34)***		97.14% (34)	
	***Not suspected of having Covid-19***	***1.56% (1)***		2.86% (1)	
	***Suspected of being a Covid-19 case***	***42.19% (27)***	93.10% (27)		
	***Identified as being a Covid-19 case***	***3.12% (2)***	6.90% (2)		
**Form of Covid observed**	***No symptoms***		***3.45% (1)***		
	***Mild form***		***68.97% (20)***		
	***Moderate form***		***27.59% (8)***		

*AAJT: Association for Helping Young Workers; PCR: Polymerase chain reaction

### Factors associated with morbidity attributable to SARS-CoV2 infection

The univariate analyses showed significant differences between the ‘Covid-19 case’ group and the control group for the following variables: age, education level, type of occupation, type of accommodation, compliance with social distancing measures, experiencing anxiety, hetero-aggressive behaviour, and level of personal hygiene. However, only ‘type of accommodation’ and ‘compliance’ with social distancing measures were significantly associated with being identified as a Covid-19 case in the multivariate analyses. The variables kept in the final multivariate model (AIC = 62.236) were ‘compliance with social distancing measures’, ‘type of accommodation’ and ‘body hygiene’(Table 6). To assess the final multivariate model, we studied other correlations and found that compliance with social distancing measures was not impacted by age. A correlation was found between education level and type of occupation. In particular, not exercising a professional activity was strongly correlated with having a junior high-school education level.

**Table 6:** Final multivariate model (Akaike information criterion = 62.24)

Variable	***Modality***	***Multivariate analysis*** ***(α = 0.05)***	***OR***	***IC***_***95%***_***OR***
**Type of accommodation**	*Single room*	*1*		
	***Single apartment***	**0.09003**	15	0,81 - 50,67
	***Shared room***	**0.47187**	0,43	2,11.10^−2^ - 3,19
	***Shared apartment***	**0.00761**	**23,45**	**3,18 - 496,79**
**Compliance with social distancing measures**	*Yes*	*1*		
	***No***	**0.02410**	**8,53**	**1,50 - 71,36**
**Body hygiene**	*Sufficient/Healthy*	*1*		
	***Insufficient/Unhealthy***	**0.99242**	1,99.10^8^	3,11.10–^76^ - ∞

The logistic regression on our final model revealed that participants living in shared accommodation were at significantly greater risk of Covid-19. They accounted for 34.48% (*n* = 10/29) of the ‘Covid-19 case’ group but only 2.86% (n = 1/35) of the control group. Failure to respect social distancing measures was also significantly associated with a higher risk of Covid-19. The odds ratios for both of these correlations were significantly greater than 1. Body hygiene also appeared in our final multivariate model, with the ‘insufficient body hygiene’ modality having an odds ratio greater than 1. However, the extent of the confidence interval of this odds ratio remained mostly around 1.(Table 6).

Also of note are close-to-significant trends for the following variables: time living in France (*p* = 0.06), wearing of a mask (*p* = 0.1), compliance with lockdown measures (p = 0.06), and regular hand washing (*p* = 0.07)(Tables 2 and 3).

## Discussion

We found that the main risk factors associated with Covid-19 infection were living in a shared apartment and not complying with social distancing measures. The first factor can be explained by the difficulty to restrict proximity within common areas. Furthermore, we know that SARS-CoV2 can stay airborne for up to three hours and survive on surfaces [28]. If a roommate is a carrier, air circulation may be sufficient to transmit the virus, even if efforts to maintain physical distancing are made. Despite this finding regarding shared apartments, multivariate results showed that sharing a bedroom was not associated with an increased risk of contracting Covid-19. This difference could be explained by the fact users living in shared bedrooms are more aware of the risk for themselves and for their roommates, and consequently make greater efforts to implement protective measures inside and outside their accommodation.

With regard to the second factor mentioned above, mass lockdown, as well as other measures - including social distancing, wearing a mask and regular hand washing - have been the pillars of the strategy to limit the spread of SARS-CoV2 in France and elsewhere [7, 29]. Our result that not complying with social distancing measures was associated with a great risk of Covid-19, reflects results in the available literature indicating that social distancing is a protective factor of SARS-CoV2 infection [30]. Although not significant in our study, wearing a mask, regular hand washing and compliance with lockdown measures are known to protect against Covid-19 [30, 31]. We found *p*-values ​​close to 0.05 for each of these measures. These values could have been even lower had we included more subjects in both study groups (i.e., more statistical power). While the concept of personal hygiene is very subjective, it nonetheless overlaps with the protective measures mentioned above [32]. Taking all these findings into consideration, we can only confirm the significant association between social distancing measures and a greater risk of Covid-19. This would therefore seem to be the most effective preventive measure for socially vulnerable populations in Marseille.

Age was a significant factor in our univariate analyses, probably because younger AAJT service users benefit more from the medical surveillance which the association offers. Indeed, younger users ask for healthcare more often and intervention teams pay more attention to this subpopulation [33]. Accordingly, younger participants were more likely to be diagnosed as potential carriers of SARS-CoV2. Compliance with social distancing measures was not associated with age. Despite the generally young age of the users, protective measures were generally understood and respected.

On the contrary, participants who were observed with hetero-aggressive behaviour tended to disregard social distancing measures. Furthermore, the AAJT healthcare team reported that some of their users found it difficult to comply with the lockdown measures. Sometimes, intervention teams had to deal with aggressive behaviour and protests. In addition, a large proportion of the participants experienced psychosocial stress linked to lockdown measures [34]. Specifically, some participants described the lockdown and the atmosphere it created as “anxiety-provoking” [14]. It may be some of the Covid-19 symptoms observed in these people had a somatic origin [35].

The AAJT services immediately isolated suspected cases, despite some having psycho-social problems. During the first lockdown, France’s healthcare system prioritized the identification and management of patients suspected of carrying SARS-CoV2 at the expense of patients wishing to consult healthcare professionals for other reasons [7]. Indeed, we observed this when comparing the different times between symptom onset and treatment for the different study groups. This urgency was justified however, by the danger identified in Covid-19 spread models [36, 37]. Nevertheless, these treatment delays were generally shorter than pre-pandemic delays for vulnerable populations, especially for users suffering from a chronic pathology or multimorbidity, and those exposed to the risk of decompensation [38].

The fact that almost all of the participants in our study were beneficiaries of state healthcare benefits ensured treatment and free screening for those who wished to be tested. In the present study, only two of the 51 people who had a PCR test were positive. However, the long delay before test availability, and the temporal discontinuity in performing them, may mean that we underestimated the number of SARS-CoV2 carriers [39]. All things considered, despite the problems raised by collective accommodation and non-compliance to social distancing measures, the spread of infection was effectively contained by the AAJT’s services and the other structures providing accommodation to vulnerable populations in Marseille. This could also explain our low rate of positive PCR results [40].

This study has limitations. First, the complexity of the ongoing pandemic context and the small number of people mobilized within AAJT’s healthcare team meant that we could not include all of the association’s users in the study. However, the diversity of the support services offered by the AAJT ensures a degree of representativeness in our sample, which in turn should have limited selection bias. Finally, the disparities we found concerning the level of certainty about SARS-CoV2 infection could be a source of classification bias. Accordingly, the results obtained should be interpreted taking this into account.

The study also has strengths, including the fact that we used a standardized questionnaire which was specifically modified to match with our study population and having the other vocation of supporting healthcare team in their diagnosis and treatment.

Marseille is the second largest city in France. Furthermore, it is a port city which has been an important passageway and place of stay for migrants for millennia [41]. The city is characterized by high levels of poverty and inequality [42]. Accordingly, studying this geographical area was necessary to propose appropriate strategic approaches to control the spread of Covid-19 in the area. This study, by highlighting the psychological, material and rent-based difficulties social associations in Marseille must face, demonstrates the vulnerability that characterizes their organizations when it comes to dealing with a pandemic. The vulnerable populations - most of whom are relatively young - which these associations provide help to are not yet on the priority list for vaccination in France. However, as our study shows, they are significant Covid-19 spreaders.

By focusing on patients affected by Covid-19, we aimed to identify the determinants of infection in socially vulnerable populations in Marseille, with a view to limiting the spread of Covid-19 while waiting for more generalized vaccination to begin.[8**,**43] In this context, the present study highlights the need to allocate more individual housing units to social structures. The collective nature of bathrooms and showers in social shelters is also an important point to consider in terms of the spread of SARS-CoV2. Implementing a strategy to ensure that only one person at a time uses these facilities - followed by disinfection - could be beneficial. Furthermore, developing psychological supports often already offered in social structures is of paramount importance. The anxiety-inducing context of the current Covid-19 crisis and the associated restrictive measures are potential triggers or aggravators for states of psychiatric and psychological disorders [14]. Given the ease of communication which community mediators have with vulnerable populations, it could be beneficial to have them present in exchanges between users and social workers and mental health specialists. Finally, social networks must be considered when it comes to communicating effectively to younger audiences [43, 44]. NGOs should be provided with larger budgets for community management, a dimension all too neglected by health authorities, funders, and sometimes by the NGOs themselves. This would help communicate vaccination campaign details when Covid-19 vaccines finally become more available, and educate younger audiences who are particularly susceptible to believing conspiracy theories [45].

## Conclusion

The fact that AAJT is a large, well-established association providing not only social support and accommodation, but also and healthcare follow-up services in Marseille on a daily basis, justified exclusively involving this organisation and its users in our study to understand emerging Covid-19 issues in the city’s vulnerable populations. Constraints linked to accommodation conditions and the very long waiting times before screening opportunities were a major concern for both the association and its users. Irrespective of how well a user implements Covid-19 protective measures, the efficacy of the latter depends on accommodation conditions. Similarly, some AAJT service users had serious and potentially deteriorating co-morbidities. Therefore, the risk inherent to collective housing justifies the attribution of individual housing for social structures. Moreover, diagnosing infected persons early means it is then possible to implement a truly effective isolation strategy and protect other users [46]. Fortunately, the NGO in Marseille - including AAJT - were able to control the spread of the virus during the first lockdown thanks to their irreproachable vigilance and speed of action. However, had there been a massive and sudden upsurge in infected numbers, the lack of available individual housing means that it would have been very hard for them to protect their users. Observing the problem through this prism, the allocation of individual housing and screening tests to structures involved with the most vulnerable populations in society would appear to be essential in the fight against the spread of SARS-CoV2. Moreover, associations must also take into account the psychological vulnerabilities and sensitivities of their service users. These dimensions further complicate the implementation of protective measures and vaccination adoption [11]. More detailed research on this situation is needed, with a focus on proposing adapted psychological care programmes and developing new digital educational systems especially for younger audiences [43].

## Supplementary Information

**Additional file 1.** Questionnaire used to collect data (English version). This is an english translation of the questionnaire developed specifically for this study.

## Data Availability

Public access to the database is closed as special authorization is required by the CNIL to consult it (*CNIL reference number: 2217364)*. With prior authorization by the CNIL, the dataset can be made available by the corresponding author on reasonable request.

## Abbreviations

SARS-CoV2: *Severe Acute Respiratory Syndrome Coronavirus 2*
Covid-19: Coronavirus Disease 2019
AAJT: Association of Aid to Young Workers
PRAPS: Regional Programme for Access to Prevention and Care for the most vulnerable populations
RT-PCR: Reverse Transcription Polymerase Chain Reaction
AME: State Medical Assistance
CNIL: National Commission for Information Technology and Liberties
WHO: World Health Organization
NSAID: Non-Steroidal Anti-Inflammatory Drugs
AIC: Akaike Information Criterion
