# Supplementary material for: Factors associated with SARS-CoV2 infection and care pathways among the most vulnerable populations living in Marseille: a case control study
Source: BMC Public Health. 2021 Sep 19;21:1704. doi: 10.1186/s12889-021-11716-6 (PMC8449995; doi:10.1186/s12889-021-11716-6)
Supplement: Supplementary file 1 — Additional file 1. Questionnaire used to collect data (English version). This is an english translation of the questionnaire developed specifically for this study. [file 12889_2021_11716_MOESM1_ESM.pdf]

Questionnaire

Date of intervention: …./…./………

For symptoms suggestive of Covid-19: ☐ Yes ☐ No

Filled by: ☐ Elise ☐ Bérangère ☐ Ismaïl

Given name initial: ……….. **Surname initial**: ……….

Service taking in charge the user :

☐ Support for young adults
☐ Social Residence for Young Workers
☐ Emergency accommodations
☐ Social Housing for Children (Diffuse)
☐ Reception Centre for Asylum Seekers
☐ Social integration facilities
☐ Social Housing for Children (Collective)
☐ Professional & social inclusion facilities

Socio-demographic characteristics

Date of birth: …./…./……… **Sex:** ☐ Male ☐ Female

Geographical area of origin: …………

Time living in France: …………

Education level:

☐ No diploma ☐ Junior high school ☐ High school ☐ University

Type of occupation:

☐ No professional activity ☐ Student ☐ Factory worker ☐ Artisan ☐ Employee

Type of accommodation: ☐ Single room ☐ Single apartment ☐ Shared room ☐ Shared apartment

Conjugal status: ☐ Single ☐ In a relationship **Number of dependent children:** …….

Social support:

☐ National health insurance cover
☐ Complementary insurance cover
☐ State Medical Assistance
☐ No social support
☐ Not available information

User recent history (from January 2020 to the onset of symptoms)

Flu-like episode occurred since January 2020: ☐ Yes ☐ No

Wearing a mask: ☐ Yes ☐ No

Understanding of confinement measures: ☐ Yes ☐ No

Compliance with lockdown measures: ☐ Yes ☐ No

Understanding of barrier measures: ☐ Yes ☐ No

Compliance with social distancing measures: ☐ Yes ☐ No

Regular hand washing: ☐ Yes ☐ No

Access to hydroalcoholic solution to wash hands: ☐ Yes ☐ No

Presence of persons suspected of having Covid-19: ☐ Yes ☐ No
If so : How many suspected people in the entourage? : …………..
Confirmed by a test: ☐ Yes ☐ No **How many people confirmed by a test? :** ……….
Type : ☐ Family members ☐ Neighbours / Roommates ☐ Friends ☐ Coworkers ☐ Social workers
How many days ago was the last risky contact? : ………

Hospitalisation since January 2020: ☐ Yes ☐ No
If so, date of hospitalisation: …./…./………

User who had tuberculosis screening in 2019/2020: ☐ Yes ☐ No

If so, result: ☐ Positive ☐ Negative

User who had psychiatric/psychological support during confinement: ☐ Yes ☐ No

Other identified health needs:

Somatic: ☐ Chronic pathology decompensation
If so : ☐ Cardiac ☐ Infectious ☐ Respiratory ☐ Psychiatric ☐ Other
Psychiatric : ☐ Anxiety ☐ Sadness ☐ Delirium ☐ Insomnia ☐ Hetero-aggressive behaviour
☐ Self-aggressive behaviour ☐ Suicide attempt ☐ Other

Addictological: ☐ Sensation of lack ☐ Request for substitution ☐ Overdose ☐ Other

Other identified needs:

☐ Insufficient/unhealthy nutrition ☐ Insufficient/unhealthy sleep ☐ Insufficient/unhealthy body hygiene
☐ Insufficient/unhealthy social life ☐ Insufficient/unhealthy physical activity

Clinical

Date of symptoms onset: …………

Fever: ☐ Yes ☐ No

Measured temperature: …..

Chills: ☐ Yes ☐ No

Asthenia: ☐ Yes ☐ No

Headaches: ☐ Yes ☐ No

Sore throat: ☐ Yes ☐ No

Dyspnea: ☐ Yes ☐ No

Difficulty speaking due to dyspnea: ☐ Yes ☐ No

Cough: ☐ Yes ☐ No

Chest pain: ☐ Yes ☐ No

Pneumonia: ☐ Yes ☐ No

Conjunctivitis: ☐ Yes ☐ No

Myalgia: ☐ Yes ☐ No

Arthralgia: ☐ Yes ☐ No

Anosmia: ☐ Yes ☐ No

Nausea and/or vomiting: ☐ Yes ☐ No

diarrhea: ☐ No ☐ Mild ☐ Moderate ☐ Severe ☐ Profuse

Skin rash: ☐ Yes ☐ No

Septic shock: ☐ Yes ☐ No

Pulse: ………… **Oxygen saturation:** ……………. **Respiratory rate:** …………….

History and comorbidities

User at risk of developing complications ?:

☐ Cardiovascular history

☐ Presence of respiratory pathology

☐ Presence of diabetes

☐ Presence of renal failure

☐ Presence of immunosuppression

☐ Presence of cancer

☐ Presence of liver disease

☐ Presence of morbid obesity

Presence of other pathologies: ☐ Yes ☐ No **How many:** ……………………..

Presence of pregnancy: ☐ Yes ☐ No

If so, stage: ☐ First trimester ☐ Second trimester ☐ Third trimester

Toxic substances/drugs use:

☐ Alcohol ☐ Tobacco ☐ Cannabis ☐ Cocaine ☐ MDMA*[1]/NSD*[2] ☐ IVDU*[3]

☐ Medicine misuse

*[1]*MDMA : 3,4-Methylenedioxymethamphetamine* ; *[2]*NSD : New synthetic drugs* ; *[3]*IVDU : Intravenous drug use*

Ongoing treatment

Long-term treatment:

☐ Biotherapy/Corticosteroid therapy ☐ Immunosuppressant ☐ Anticancerous chemotherapy

☐ Antihypertensive

Covid self-medication (other than paracetamol): ☐ Yes ☐ No

If so : ☐ Chloroquine ☐ Lopinavir ☐ Others : _____

Recent intake of NSAIDs*[4]: ☐ Yes ☐ No

*[4]*NSAIDs : Non-steroidal anti-inflammatory drugs*

Care of a patient not having symptoms suggesting COVID-19

Motif de consultation:

☐ Follow-up of a chronic pathology ☐ Pain management ☐ Infectious disease

☐ Gastroenterology ☐ Psychology/Psychiatry ☐ Gynaecology/Obstetrics

☐ Dermatology ☐ Orthopaedics ☐ Ophthalmology

☐ Other: ....

Care of a patient having symptoms suggesting COVID-19

Test performed: ☐ Yes ☐ No

Test result: ☐ Positive ☐ Negative

Chest scan: ☐ Suggesting COVID-19 ☐ Not suggesting COVID-19 ☐ Not performed

Strict self-isolation: ☐ Yes ☐ No

Compliance with self-isolation: ☐ Yes ☐ No

Denial about the possibility of being Covid-19 positive: ☐ Yes ☐ No

Modification of accommodation for the user: ☐ Yes ☐ No

If so: ☐ Shared apartment ☐ Single room ☐ Shared room ☐ Single apartment

Hospitalisation: ☐ Yes ☐ No

Treatments: ☐ Yes ☐ No

Paracetamol: ☐ Yes ☐ No

Antipyretic: ☐ Yes ☐ No

Anti-emetic: ☐ Yes ☐ No

Oxygen: ☐ Yes ☐ No

Others: ……….

Medical follow-up

Follow-up D1: ☐ Yes ☐ No

If so:

Temperature>38: ☐ Yes ☐ No

Dyspnea: ☐ Yes ☐ No

Oxygen saturation: …….. **Respiratory rate:** ……..

Compliance with self-isolation: ☐ Yes ☐ No

Hospitalisation: ☐ Yes ☐ No

Medical opinion: ☐ Yes ☐ No

Symptoms have disappeared or have greatly decreased: ☐ Yes ☐ No

Follow-up D3: ☐ Yes ☐ No

If so:

Temperature>38: ☐ Yes ☐ No

Dyspnea: ☐ Yes ☐ No

Oxygen saturation: …….. **Respiratory rate:** ……..

Compliance with self-isolation: ☐ Yes ☐ No

Hospitalisation: ☐ Yes ☐ No

Medical opinion: ☐ Yes ☐ No

Symptoms have disappeared or have greatly decreased: ☐ Yes ☐ No

Follow-up D5: ☐ Yes ☐ No

If so:

Temperature>38: ☐ Yes ☐ No

Dyspnea: ☐ Yes ☐ No

Oxygen saturation: …….. **Respiratory rate:** ……..

Compliance with self-isolation: ☐ Yes ☐ No

Hospitalisation: ☐ Yes ☐ No

Medical opinion: ☐ Yes ☐ No

Symptoms have disappeared or have greatly decreased: ☐ Yes ☐ No

Follow-up D9: ☐ Yes ☐ No

If so:

Temperature>38: ☐ Yes ☐ No

Dyspnea: ☐ Yes ☐ No

Oxygen saturation: …….. **Respiratory rate:** ……..

Compliance with self-isolation: ☐ Yes ☐ No

Hospitalisation: ☐ Yes ☐ No

Medical opinion: ☐ Yes ☐ No

Symptoms have disappeared or have greatly decreased: ☐ Yes ☐ No

Follow-up D14: ☐ Yes ☐ No

If so:

Temperature>38: ☐ Yes ☐ No

Dyspnea: ☐ Yes ☐ No

Oxygen saturation: …….. **Respiratory rate:** ……..

Compliance with self-isolation: ☐ Yes ☐ No

Hospitalisation: ☐ Yes ☐ No

Medical opinion: ☐ Yes ☐ No

Recovery: ☐ Yes ☐ No

Sequelae: ☐ Yes ☐ No

If so, which ones ?: ……..

Death: ☐ Yes ☐ No

If so, date of death: __/__/____

Lost to follow-up: ☐ Yes ☐ No

If so, on how many days from the onset of symptoms ?: ……..

Biological

Serodiagnosis: __/__/____

Presence of immunoglobulins M: ☐ Yes ☐ No

Presence of immunoglobulins G: ☐ Yes ☐ No

PCR test: __/__/____

Result: ☐ Positive ☐ Negative

Conclusion

COVID-19 diagnosis: ☐ Confirmed ☐ Suspected ☐ Not suspected ☐ Not having Covid-19

If the patient is a carrier or potentially a carrier, what is the observed form of COVID-19 ?:

☐ Asymptomatic ☐ Benign ☐ Moderate ☐ Severe
